# Poisons

**Published:** 1885-08

**Authors:** 


### Poisons.

From a Latin word, meaning "drink," as poisons are generally taken in that way; and are either "corrosive," such as destroy or kill the texture of the part; or "constitutional," affecting the system, through the nerves and blood-vessels. Mineral and acid poisons, as lead, copper, arsenic, oxalic-acid, aqua-fortis, and the like, kill the living parts on the instant of touching, and death speedily results, from inflammation, swelling, and mortification.

Alcohol, opium, prussic acid, strychnine, and the like, are constitutional, and affect the system through the nerves and blood-vessels. There are, besides the gases, over sixty solid substances, in nature, which destroy life in a day, an hour, a minute. An "antidote" is that which instantly renders a poison innocuous by removal or chemical combination. For corrosive poisons, such as mineral and acid, indicated certainly by the patient carrying the hand to the throat, swallow instantly sweet oil, train-oil, or any other simple oil or grease first at hand. This soothes, protects, and vomits; or take magnesia, soap, or saleratus, in water.

As to the constitutional poisons, instant removal is imperative; and the very best thing in all nature. as well as most generally at hand, is a heaping teaspoonful each of common salt and ground mustard, stirred quickly in a glass of cool or warm water, and swallowed on the spot. This usually causes instantaneous vomiting. As soon as this ceases, as there may be some of the poison left in the stomach, swallow the white of an egg or two; and to make assurance doubly sure, drink most freely of very strong coffee. The egg is best for corrosive poisons; the coffee, for the constitutional. A quart of very strong cold coffee should be put away in a bottle in every family, for such uses; especially as it is the antidote for a a larger number of poisons than any other substance in nature.

The above are intended as expedients, to be employed while a physician is being procured.

